# Structure of the Flight Muscle Thick Filament from the Bumble Bee, *Bombus ignitus*, at 6 Å Resolution

**DOI:** 10.3390/ijms24010377

**Published:** 2022-12-26

**Authors:** Jiawei Li, Hamidreza Rahmani, Fatemeh Abbasi Yeganeh, Hosna Rastegarpouyani, Dianne W. Taylor, Neil B. Wood, Michael J. Previs, Hiroyuki Iwamoto, Kenneth A. Taylor

**Affiliations:** 1 Institute of Molecular Biophysics, Florida State University, Tallahassee, FL 32306-4380, USA; 2 Department of Molecular Physiology & Biophysics, University of Vermont, Larner College of Medicine, Burlington, VT 05405, USA; 3 Scattering and Imaging Division, Japan Synchrotron Radiation Research Institute, SPring-8, Hyogo 679-5198, Japan

**Keywords:** cryoelectron microscopy, myosin, coiled coil, striated muscle, asynchronous flight muscle

## Abstract

Four insect orders have flight muscles that are both asynchronous and indirect; they are asynchronous in that the wingbeat frequency is decoupled from the frequency of nervous stimulation and indirect in that the muscles attach to the thoracic exoskeleton instead of directly to the wing. Flight muscle thick filaments from two orders, Hemiptera and Diptera, have been imaged at a subnanometer resolution, both of which revealed a myosin tail arrangement referred to as “curved molecular crystalline layers”. Here, we report a thick filament structure from the indirect flight muscles of a third insect order, Hymenoptera, the Asian bumble bee *Bombus ignitus*. The myosin tails are in general agreement with previous determinations from *Lethocerus indicus* and *Drosophila melanogaster*. The Skip 2 region has the same unusual structure as found in *Lethocerus indicus* thick filaments, an α-helix discontinuity is also seen at Skip 4, but the orientation of the Skip 1 region on the surface of the backbone is less angled with respect to the filament axis than in the other two species. The heads are disordered as in *Drosophila*, but we observe no non-myosin proteins on the backbone surface that might prohibit the ordering of myosin heads onto the thick filament backbone. There are strong structural similarities among the three species in their non-myosin proteins within the backbone that suggest how one previously unassigned density in *Lethocerus* might be assigned. Overall, the structure conforms to the previously observed pattern of high similarity in the myosin tail arrangement, but differences in the non-myosin proteins.

## 1. Introduction

Among the several kinds of filaments that make up striated muscle, the thick, myosin-containing filaments are perhaps the least understood. The arrangement of myosin heads in relaxed thick filaments was not clarified until 2005 [1], which showed the myosin heads arranged in the same folded head conformation found in the ATPase inhibited form of smooth muscle myosin [2], now known as the interacting heads motif (IHM). Nearly all thick filament structures obtained at 25 Å resolution or higher have shown the heads arranged in this motif [3,4,5,6,7,8], which has become associated with the super relaxed state of muscle [9]. The motif has also been observed in myosin isolated from a wide range of cells and tissues and appears ubiquitous in the myosin II of nearly all cells that have a multicellular life-cycle stage [10].

The IHM has a roughly planar structure, with the actin-binding domain of the Free Head extending perpendicular to this plane [2]. The actin-binding interface of the other head, the Blocked Head, abuts the Free Head and lies roughly within the plane. Most thick filaments, whose structures have been solved at sufficient resolution to resolve the individual myosin heads, show the normal vector of the plane of the IHM oriented approximately along a radial vector emanating from the thick filament backbone, utilizing a predominately charged interaction between the Blocked Head, with the initial segment of the myosin coiled coil known as the proximal S2 [11,12] and including interactions with neighboring motifs to stabilize the structure [1,5]. Recently, the thick filament structure from the flight muscles of the large waterbug *Lethocerus indicus* showed a novel orientation with the normal vector to the plane of the IHM oriented approximately parallel to the filament axis [8]. No interaction occurs between the myosin heads and the proximal S2 or with neighboring IHMs. Rather than having the proximal S2 interaction holding the IHM against the filament backbone, the Free Head binds tangentially against the backbone with the Blocked Head, oriented as if to “pin” the Free Head in place.

The relaxed thick filament reconstructions from *Drosophila melanogaster* failed to show any ordered myosin heads [13,14] leaving open the question of whether this particular muscle forms an IHM that is itself disordered, because it fails to attach to the filament backbone, or whether the motif forms at all. Supporting the former was the presence of non-myosin proteins decorating the filament backbone in such a way that they could provide a steric block to the binding of myosin heads. Supporting the latter is the general difficulty in persuading *Drosophila* flight muscle myosin II to form the IHM in vitro [10].

Here, we report the subnanometer structure of thick filaments from the bumblebee *Bombus ignitus* of the insect order Hymenoptera. The *B. ignitus* thick filament backbone conforms generally to the pattern observed in the dipteran *Drosophila melanogaster* and the hemipteran *Lethocerus indicus* but with some differences. No non-myosin proteins that conform to the imposed helical symmetry were found decorating the outside surface of the filament backbone. The arrangement of myosin tails is very similar with one notable departure. The non-myosin protein flightin reveals a newly resolved extension at the C-terminus that overlaps a previously unassigned density in both *Drosophila* and *Lethocerus*. Density is found in the core of the filament indicating a higher amount of paramyosin than is found in *Drosophila* and more comparable to that found in *Lethocerus*.

## 2. Results

**Figure 1:**
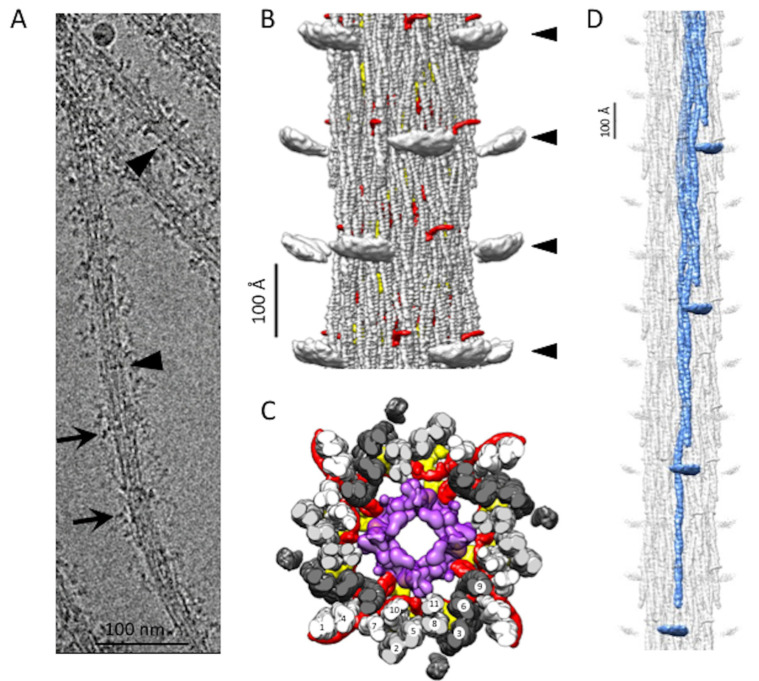
*Bombus* thick filament reconstruction. (**A**) An electron micrograph of a thick filament. The longitudinal lines in the thick filament backbone are produced by the coiled-coil domains of myosin and paramyosin. The disordered dark transverse densities marked by black arrows are possibly the myosin heads. Occasionally, transverse densities (arrowheads) are observed, which are reminiscent of the Free Heads in *Lethocerus* thick filaments [8]. (**B**) The reconstruction shows three full crowns of the *Bombus* thick filament. The “floating” densities (arrowheads) are the average positions of the myosin heads when helical and 4-fold symmetry are enforced. Non-myosin density, flightin (red) is visible extending from the backbone surface. (**C**) Transverse view through one crown showing the relative positions of flightin (red), myofilin (yellow), putative paramyosin (purple) and the curved layers (dark gray, light gray and white). Note that both flightin and myofilin have folded domains on the inside surface of the annulus of myosin tails that could contact paramyosin. (**D**) The 12-crown extended filament, with one curved layer highlighted in blue. The floating densities are not connected to the myosin tail due to the disorder in the proximal S2.

Electron micrographs of *Bombus* thick filaments show longitudinal stripes from the ordered arrangement of the coiled-coil of myosin tails and paramyosin but generally do not show evidence of ordering of the myosin heads (Figure 1A). All filament segments for reconstruction were picked manually and 2-D classified with single-particle processing methods. We computed the 3D density map (Figure 1B,C) from ~110,000 filament segments ~845 Å in length, subsequently boxed out and processed using cisTEM [15]. The best-estimated resolution of the reconstruction computed by FSC and reported by cisTEM is 4.34 Å. We repeated the gold standard FSC using the FSC validation in CryoSPARC [16] and obtained a result of 4.82 Å (Supplemental Figure S1A). The local resolution estimation in CryoSPARC [16] calculated from a map with the floating densities representing the disordered myosin heads masked out returned a resolution within the backbone between 6–7 Å (Supplemental Figure S1B). The overall resolution computed by CryoSPARC is reduced by the comparatively poor order in the paramyosin core. We estimate the map resolution to be 6 Å by comparison to the *Lethocerus* flight muscle thick filament map that also achieved a similar resolution [8]. The α-helices of the *Bombus* myosin tails are clearly separated in the sharpened map, although the residue side chains are not generally resolved.

### 2.1. Myosin Tail Structure and Arrangement

The *Bombus* thick filament backbone has many features in common with the backbones of both *Drosophila* and *Lethocerus*. Myosin tails of *Bombus* are contained within an annulus with an outer diameter of ~190 Å and an inner diameter of ~80 Å, which is similar to *Lethocerus* [8] but slightly larger than *Drosophila* [13]. Of the initial references tried, the low pass filtered *Lethocerus* map generated a better reconstruction than using the *Drosophila* map or starting from a structure-free reference. This may be due to the better match of diameter with that of *Lethocerus*. In addition, the ordered heads of *Lethocerus* may have been better suited to achieving an initial alignment of the 145 Å axial spacing. Except for the disordered myosin heads (Figure 1B), the relaxed *Bombus* thick filament appears more similar to *Lethocerus* than *Drosophila* in overall structure, particularly in the relatively featureless outer surface (Figure 1B). The top view of the averaged 3D image of the *Bombus* thick filament shows a dense core, which, based on the *Lethocerus* thick filament, is probably paramyosin in composition (Figure 1C).

In *Bombus*, the myosin tails are arranged in “curved molecular crystalline layers” (Figure 1D), which we will abbreviate as “curved layers” to distinguish them from the ribbon-like smooth muscle thick filaments that inspired Squire’s original model of the thick filament backbone [17]. Each curved layer is made of three myosin coiled-coil tails, each offset axially by three crowns (1 crown = 145 Å). Three curved layers make up the asymmetric unit. Each myosin tail is slightly shorter than 11 crowns (1600 Å) so that a transverse slice through any curved layer always intersects three tails but, at one out of three crowns, intersects four tails (Figure 1C). The 11th crown of the myosin tail is the proximal S2, which lies outside of the backbone and is largely disordered in *Bombus* at 6 Å resolution at the contour cutoff that best displays the coiled coils in the myosin tail annulus. A transverse slice through the entire backbone cuts through 12 curved layers in *Lethocerus*, *Drosophila*, and *Bombus*.

The myosin tail can be separated by proteolysis of myosin monomers in high salt into two parts, Subfragment 2 or S2 and Light MeroMyosin or LMM [18]. Except for a structureless non-helical C-terminal segment, the myosin tail is structured as a parallel, 2-stranded, α-helical coiled-coil. We resolved the 10 crowns of the myosin tail structure in *Bombus* and a short stub of the proximal S2 (Figure 1B–D). The remainder of the proximal S2 that connects to the heads was not visible at this contour cutoff. How much proximal S2 is visible depends on the chosen contour threshold and low pass filter cutoff. As the threshold and resolution are lowered, more S2 is visible, indicating that its mobility increases as it approaches the floating density. However, at any reasonable threshold or resolution cutoff that can still reveal the coiled-coil twist, the proximal S2 density points directly at the floating densities (Figure 1D) but does not reach them. The helical symmetry as determined by Relion [19] was very similar to the three insect species for which we have determined structures (Table 1). *Bombus* has a slightly smaller axial repeat and a slightly higher helical angle. The minor difference in axial rise compared with the 145 Å determined from the X-ray fiber diffraction is likely due to a magnification error. We, therefore, scaled the pixel size to enforce the 145 Å determined by the X-ray. The differences in helical angle are small and possibly within experimental error.

### 2.2. Coiled Coils and Skip Residues

**Figure 2:**
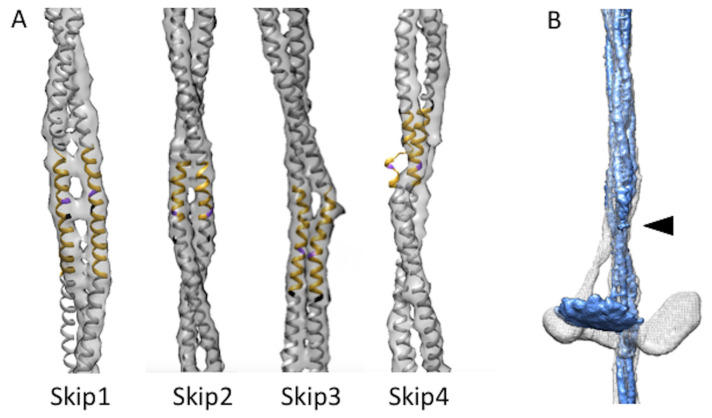
Skip residues and proximal S2. (**A**) Skip residue accommodation regions of *Bombus ignitus* (grey map envelope) are well aligned with the *Lethocerus indicus* atomic model (PDB–7KOG) shown in yellow with skip residue colored in purple for the accommodation region and white elsewhere [21], except for Skip 1 where the *Lethocerus* atomic model falls outside the *Bombus* envelope due to the latter’s different azimuthal rotation when compared with *Lethocerus* (white). (**B**) Superposition of curved layers from *Lethocerus* (white) and *Bombus* (blue). When the backbone is low pass filtered to the same resolution that reveals the average myosin head position additional proximal S2 is revealed up to the arrowhead. The *Lethocerus* proximal S2 (white) prominently bends away from the trajectory of the coiled coils within the backbone as a consequence of Free Head binding to the filament backbone. In *Bombus*, the proximal S2 (blue) follows the trajectory of the backbone-embedded tail downwards at least as far as the resolved density shown. The *Lethocerus* density map has been low pass filtered to 7 Å resolution to make it comparable to the displayed *Bombus* map.

We used rigid-body fitting in Chimera [20] to compare the four *Bombus* myosin tail segments containing skip residues with the corresponding segments in the atomic model of the *Lethocerus* flight muscle myosin tail [21] (Figure 2A). Skips 2 and 3 of both atomic models aligned well. Skip 3 showed the same distinct kink in one helix within the accommodation region where flightin passes between the myosin tails of one curved layer. In *Bombus*, the flightin WYR domain physically contacts the curved layer and is partially inserted into the gap made by the kink in the Skip 3 accommodation region and the neighboring tail. Without Skip 3, the WRY domain cannot nestle between the rods, but the extended polypeptide chain of flightin still can pass through the curved layers at both ends. Skip 4 showed a thin density resembling a loop in one chain, but in a different orientation to that seen in *Lethocerus*. The other chain appears to be a continuous α-helix, as seen in *Lethocerus*. Near the Skip 4 region, the C-terminal segment of flightin passes between a pair of myosin tails, possibly causing the loop to form in the Skip 4 region of one chain. The most significant difference is seen in Skip 1. The accommodation region of Skip 1, which is where the coiled-coil changes structure to parallel α-helices in order to accommodate the inserted amino acid residue, in *Lethocerus* consists of ~30 residues of parallel α-helices angled significantly with respect to the filament axis [21]. The Skip 1 accommodation region in *Bombus* is significantly less angled relative to the filament axis than in *Lethocerus*.

Where the proximal S2 enters the filament backbone, more of the S2 is visible in *Bombus* than was seen in *Drosophila* [13]. Compared with *Lethocerus*, where the proximal S2 is angled with respect to the filament axis stabilized by the Free Head binding the filament backbone, the stub of S2 seen in both *Drosophila* and *Bombus*, is parallel with the initial S2 segment as it enters the filament backbone. Only a small stub of the proximal S2 is seen outside the backbone, which points directly towards the “floating” density (Figure 2B; Supplemental Video S1).

### 2.3. Myosin Heads

**Figure 3:**
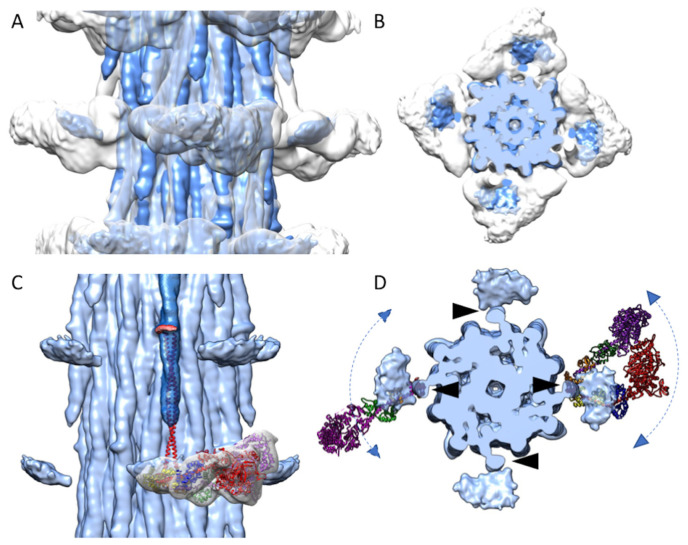
Disordered myosin heads of *Bombus* compared with those of *Lethocerus*. The *Bombus* density map (blue) is superimposed on the *Lethocerus* density map (grey) in both side view (**A**) and top view (**B**). (**C**) An atomic model of the IHM (Blocked Head heavy chain, red; Essential Lght Chain (ELC), blue; Regulatory Light Chain (RLC), yellow; Free Head heavy chain, purple; ELC, green; RLC, orange) along with the myosin head density of the *Lethocerus* reconstruction is shown. The floating densities from *Bombus* (blue) represent the average position of otherwise highly disordered myosin heads. The crystal structure of cardiac S2 (red) is aligned to the *Bombus* S2 density. The S2 atomic model points directly at the floating myosin head density. Note that the *Lethocerus* features (map and atomic model) are aligned to the cardiac S2 atomic model and not to their position in the relaxed *Lethocerus* thick filaments. (**D**) Axial view with atomic models of the Free Head (left) and IHM (right) from relaxed *Lethocerus* thick filament superimposed on the *Bombus* density map with the N-terminal domain of the RLC under the proximal S2. The position of the proximal S2 (black arrowheads) is juxtaposed with the inner edge of the floating density. The atomic models serve to illustrate how a floating density might be explained by mostly azimuthal movements of either individual myosin heads or interacting heads motifs. The floating density in *Bombus* would appear to mostly represent the average position of the disordered RLC.

The reconstruction has no density that resembles either individual myosin heads or the IHM. Apparently, the myosin heads are disordered in relaxed *Bombus* thick filaments similar to how they are in relaxed *Drosophila* flight muscle thick filaments [13]. When the reconstruction is low-pass filtered to 15 Å, a pancake-shaped floating density is observed, centered at the same radius and axial position of the ordered heads of relaxed *Lethocerus* flight muscle thick filaments but much smaller than the expected volume of an individual myosin head (Figure 3A,B). Its mass is centered at a distance of ~30 Å from the thick filament surface. Its volume at the contour threshold shown is 69,190 Å^3^, corresponding to a mass of 56 kDa. The floating density has an approximately ellipsoidal shape with the longest axis oriented azimuthally and the shortest axis oriented approximately axially. It is tilted slightly toward the bare zone. The position is essentially identical to that found in *Drosophila* [13]. The small stub of the proximal S2 points directly at the inner edge of the floating density (Figure 3C,D).

When the atomic model of cardiac S2 (red in Figure 3C; PDB: 2FXM) [22] is fit to the *Bombus* S2 density, and the myosin head density and IHM atomic model from the relaxed *Lethocerus* thick filaments are aligned to the *Bombus* S2 (Figure 3C), the cardiac S2 points at the *Lethocerus* IHM RLCs. From the top view (Figure 3D), the *Bombus* floating head density is largely overlapped with the RLCs. In the relaxed *Lethocerus* thick filament, the Free Head binds the filament backbone and in doing so displaces the proximal S2 azimuthally by 17°. Consequently, the IHM of *Lethocerus* does not align with the *Bombus* floating head density (Figure 3D). There are two possible interpretations.

Possibly, the myosin heads form an IHM, but the motif itself, lacking any stabilizing connection to the thick filament backbone, is disordered. If this were true, the motif would be moving azimuthally, axially, and radially about the short, ~11 nm length of proximal S2 (Figure 3D, right). Alternatively, the IHM does not form, and the individual heads are disordered (Figure 3D, left). Although the proximal S2 is disordered at higher resolutions, the fact that increasing amounts are visible at a lower resolution is consistent with the motion of the heads about the head-tail junction which itself is moving at the end of a comparatively stiff rod-shaped structure, the proximal S2, that is pivoting about a fixed point where it exits the filament backbone. Motions of the S2 are restricted to bending about the axis connecting its two polypeptide chains, which rotates with the pitch of the coiled-coil [21,23]. This axis is oriented radially as the proximal S2 exits the filament backbone. Thus, azimuthal movements of the heads will be larger than radial and axial movements which occur with a shorter axis.

Myosin heads are ~190 Å in length so that the large motor domain would be furthest from the pivot point, the head-tail junction (Figure 3D). If the disordered head is pivoting around the head-tail junction, its largest mass, the motor domain, will be distributed over a larger volume than its RLC, which has a lower mass but is distributed over a smaller volume relative to its size. If the IHM is not formed, then the individual myosin heads would be even more mobile while still pivoting about their connection to the proximal S2. That the floating density has a flattened, ellipsoidal shape rather than a spherical shape suggests the head motion is not equally probable in all directions.

### 2.4. Non-Myosin Densities

Non-myosin densities threading among the myosin tails are the most distinctive feature of the insect flight muscle thick filaments so far imaged at subnanometer resolution. Non-myosin proteins of *Lethocerus* and *Drosophila* are well established, but those of *Bombus* are less so, particularly for *Bombus ignitus*. All invertebrate thick filaments appear to contain paramyosin, though the amounts appear to be quite variable [24]. Two other proteins, flightin and myofilin, have been found in *Lethocerus* and *Drosophila* thick filaments and a third, stretchin-klp, has been identified in reconstructions of *Drosophila* [13,14] but is not seen in *Lethocerus*. Other proteins such as projectin, kettin, and obscurin are present within flight muscle thick filaments, but their quantities are either too low relative to myosin to show up in a helically averaged 3-D image reconstruction, or they are located in the bare zone, or the filament ends which are areas that are avoided when selecting segments for reconstruction.

For the two major proteins found among the myosin tails, flightin and myofilin, the segmentation in *Bombus* was not always clear as their assumed densities appeared to contact each other in one location, leaving the interpretation ambiguous. The segmentation was initially made based on their similarity to *Lethocerus*. The most ambiguous feature is the location where the putative flightin and myofilin densities cross. For the reason that the flightin density is well defined in *Lethocerus* and *Drosophila*, we initially believed that this density was part of myofilin. However, the continuity of the flightin density, sequence alignment, and the atomic model acquired using AlphaFold2 [25] suggested otherwise.

#### 2.4.1. Flightin 

**Figure 4:**
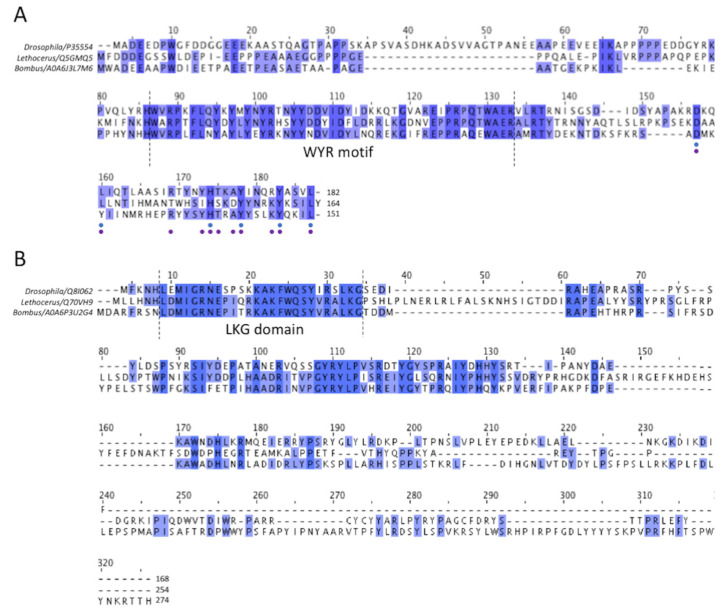
Multiple sequence alignment of flightin and myofilin of *Drosophila*, *Lethocerus* and *Bombus* flight muscle. (**A**) The alignment of flightin shows the highly conserved “WYR” motif, which probably corresponds to the common globular flightin density seen in 3-D image reconstructions of thick filaments from the three species. Comparatively, the N- and C- termini are less conserved, although some improvement in conservation is seen at the C-terminus. Among all 112 flightin sequences available in Uniprot, both predicted and observed experimentally, 6 out of 32 C-terminal residues have over 60% similarity (indicated as blue dots below), and if the entries are narrowed down to endopteryogota taxonomy, a total of 80 flightin sequences, 11 out of 32 have high similarity of over 60% (indicated as purple dots below). (**B**) For myofilin, the N-terminal sequence is well conserved, as is the shape and position of the folded domain seen among the three species, which suggests that they represent the same entity. Conservation decreases after the N-terminal domain, improves somewhat after about 30 residues in *Bombus*, and becomes very poor toward the C-terminus suggesting that the highly variable parts of the putative myofilin density represent these less well-conserved other segments of the sequence.

Of the three asynchronous flight muscle thick filaments that have been imaged at the subnanometer resolution, all have densities that can be assigned to the protein flightin based on the fact that flightin has an N-terminal segment that extends outside of the filament backbone and is thus accessible to antibody binding [26] and, when deleted, indicates a role in stretch activation through the binding to the actin [27]. Based on our sequence alignment (Figure 4A), the flightin isoforms have different lengths with that of *Drosophila melanogaster* flight muscle being the largest of the three having a length of 182 residues, predicted molecular weight 20,656 Da, with a 22 residue insert after residue 32 which is not present in *Lethocerus indicus* or those *Bombus* species whose flightin sequence has been reported. *Lethocerus indicus* flightin has a length of 164 residues and a predicted molecular weight of 19,274 Da. The sequence of *Bombus ignitus* flightin has not been reported but the flightin sequences for the four reported *Bombus* species are each 151 residues long with predicted molecular weights of 18,147 to 18,210 Da, here we used *Bombus vosnesenskii* (detected by mass spectrometry) for the sequence alignment. All four *Bombus* species have an eight-residue deletion following residue 44 and a five-residue deletion following residue 119 that are not present in either *Drosophila* or *Lethocerus*.

A well-conserved region in the middle is present in all three sequences and has been dubbed the WYR motif [28]. The flightin N-terminus lies outside of the filament backbone and is disordered. The C-terminus is embedded within the backbone based on evidence that deletion of the C-terminus results in filaments of irregular length, whereas deletion of the N-terminus produces filaments of normal length but in flies that cannot fly [27].

**Figure 5:**
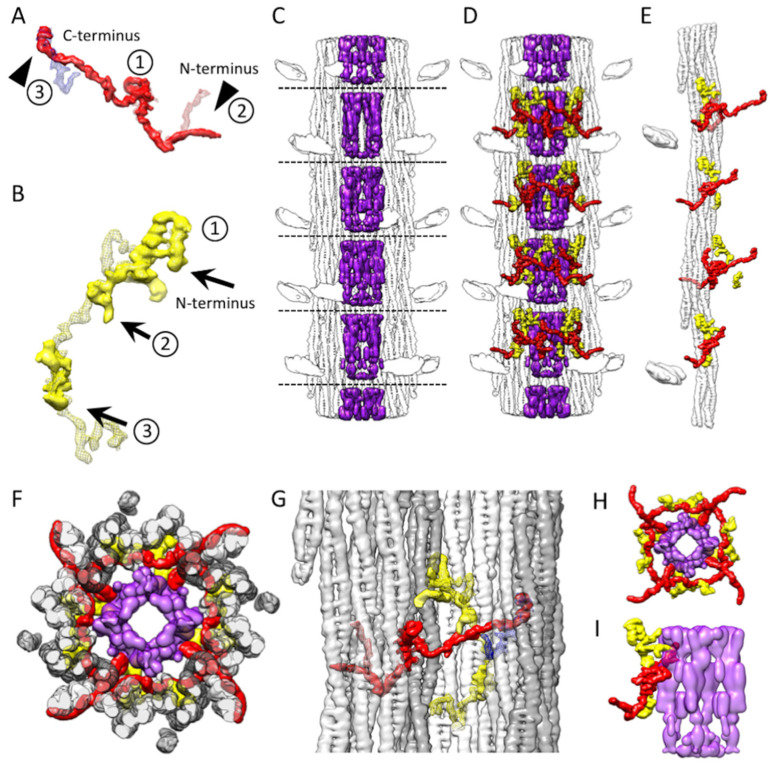
Three non-myosin densities in *Bombus* thick filament. (**A**) *Bombus* flightin molecules (red, solid) are similar among all three species with a small folded domain (labeled 1) in the middle corresponding to the WYR domain [28], a partially ordered extension outside of the filament backbone (labelled 2) on one side of the WYR domain and an extension on the other side (labelled 3). At the N-terminus (labelled 2) *Lethocerus* (red, mesh) shows the largest extension outside the filament backbone, stabilized by contacts to its proximal S2 [8]. *Drosophila* has the same feature but with a shorter extension [13] corresponding to only the overlap between *Lethocerus* and *Bombus*. The flightin N-terminal extension seen in *Bombus* leaves the backbone density at a different angle (solid red surface). The flightin C-terminus (labelled 3) overlaps a density (blue, mesh) from a *Lethocerus* thick filament reconstruction. (**B**) *Bombus* myofilin (yellow, solid) shows a novel structure, with two densities, that overlap and contact the *Lethocerus* myofilin density (yellow, mesh). (**C**) The *Bombus* paramyosin (purple, solid) in the central core. (**D**) The distribution of all three non-myosin proteins in the context of the thick filament. (**E**) Flightin and myofilin decorating a single curved layer. (**F**) The top view of a full crown slab containing all three non-myosin proteins. (**G**) Side view showing the relationship of the three non-myosin proteins to the five curved layers colored white, light gray and dark gray. (**H**) Top view and (**I**) side view showing the relationship of flightin and myofilin with the paramyosin core. Both myofilin and flightin appear to contact paramyosin.

A density corresponding in size, shape and location to features seen in both *Drosophila* [13] and *Lethocerus* [8] is seen also in Bombus (Figure 5A). This feature, about 45 residues in size for *Bombus*, was preliminarily identified as the protein flightin [8]. A larger *Bombus* flightin density is visible at the same contour threshold used in the previous reconstructions. The visible part of *Bombus* flightin corresponds to a volume (8957 Å^3^) that is larger than that of the *Lethocerus* (4099 Å^3^) and *Drosophila* (3917 Å^3^).

The density that we believe corresponds to the *Bombus* WYR motif, residues 56–102, appears nearly identical in shape to the similar density in *Lethocerus* and *Drosophila* positioned at the same location when the different reconstructions are aligned (Figure 5A; labelled 1). In *Drosophila* and *Lethocerus*, the putative WYR motif is found at one end of the flightin density. For *Bombus*, it is located in the middle of the flightin density, which would correspond to its position in the amino-acid sequence. Thus, the conservation of the WYR motif sequence and the shape of the likely WYR motif density strongly suggest correspondence.

The N-terminal side of the WYR motif has a very similar shape up to the point where the *Drosophila* density ends just beyond the outer surface of the thick filament backbone. All three have an abrupt change in direction by ~90° before exiting the thick filament backbone. The *Bombus* flightin density is visible for 38 Å after exiting the filament backbone, which is more than is seen in *Drosophila*. Beyond the short stub of density seen in *Drosophila*, the flightin density of *Lethocerus* and *Bombus* diverge with *Lethocerus* contacting the proximal S2 (Figure 5A, labelled 2). In *Lethocerus*, the proximal S2 is bent 17° azimuthally due to the binding of the Free Head to the thick filament backbone [8]. Due to the disordering of the myosin heads, this azimuthal bending is not observed in *Bombus*. When the *Bombus* contour threshold is lowered to reveal more of the proximal S2 (see for example Figure 3C) *Bombus* flightin has a contact roughly from about residue 965 to residue 971 of the myosin tail. Thus, it is possible that the flightin of both *Lethocerus* and *Bombus* are stabilized after exiting the thick filament through contact with the proximal S2.

What distinguishes *Bombus* flightin from that of *Lethocerus* and *Drosophila* is a slim, continuous, and elongated density extending toward the presumptive C-terminus (Figure 5A, labelled 3; Supplemental Video S2). The end of this feature, so far seen only in *Bombus*, overlaps well with part of the so-called “blue” density of *Lethocerus* [8] and *Drosophila* [13]. This perhaps explains why no separate density corresponding to the “blue” density is seen in *Bombus*; its “blue” density is part of flightin. The overlap between *Bombus* flightin and the *Lethocerus* “blue” density occurs at the very end of the flightin density. The flightin amino acid sequence is more conserved at the C-terminus than in the region separating it from the WYR motif, where *Bombus* flightin has a five-residue deletion (Figure 4A) that possibly explains the poor overlap with the entire “blue” density. The flightin density crosses over a feature that appears to be part of another non-myosin protein, myofilin, (Figure 5B; labelled 2). This contact occurs close to the WYR domain and may have stabilized this previously unresolved segment of flightin. The assignment of this long extension to flightin is supported by modeling the structure using AlphaFold2 (see below).

The density corresponding to the residues on the N-terminal side of the WYR motif of *Bombus* flightin, similar to that of *Lethocerus* and *Drosophila*, passes between the myosin tails of one curved layer to reach the outside of the backbone. The WYR motif itself is situated at the inside of the myosin tail annulus, where it contacts two curved myosin layers. The density on the C-terminal side of the WYR motif passes across a pair of myosin tails of one curved layer and ends up contacting yet another curved layer on the N-terminal side (Supplemental Video S4). Flightin’s interaction with five different curved layers possibly explains its impact on determining filament length [29]. Most of the myosin-flightin interactions occur either via flightin’s passage between the myosin tails within curved layers or contacts with myosin tails at the edge of the curved layer. An additional stabilizing contact, so far seen only in *Bombus*, may be with myofilin over which it crosses during this passage. The former “blue” density may have appeared as a separate entity in the previous reconstructions from *Lethocerus* and *Drosophila* because their flightin sequences are slightly longer than this region of *Bombus* flightin and possibly more mobile.

#### 2.4.2. Myofilin 

The other large non-myosin density among the myosin tails is assumed to be myofilin (Figure 5B). The sequence of *Bombus ignitus* myofilin is not available, so we will use the sequence from *Bombus terrestris*, isoform 1, which has 274 residues and a molecular weight of 32,629 Da. Other *Bombus* species with isoform 1 also have 274 residues and molecular weights ranging from 32,601–32,609. *Bombus* isoform 2 myofilin sequences have 251 residues with molecular weights that range from 29,665–29,693. The only difference between the two *Bombus* isoforms is a 23-residue C-terminal extension on isoform 1. Among the three species, *Drosophila* myofilin has the shortest sequence of 168 residues. The *Lethocerus* sequence has 254 residues with two large insertions relative to *Bombus* and *Drosophila* from residue 37 to residue 58 and residue 145 to residue 167 (Figure 4B). All three have a highly conserved N-terminal domain. Another relatively conserved sequence region occurs after the N-terminal domain but cannot be identified unambiguously in the density map. The rest of the sequence is poorly conserved as it nears the C-terminus.

Comparing the myofilin density among the three species reveals a high similarity in one of the folded domains (Figure 5B, labelled 1) seen at one end of the density. The N-terminus of the myofilin sequence among the three species is the only well-conserved region. Of the first 34 residues of *Bombus* myofilin, 19 are identical between the three species (Figure 4B). The sequence has three lysines and two each of glycine and leucine. The sequence LKG starting at *Bombus* residue 32 is absolutely conserved among 51 myofilin sequences we aligned that had “Evidence of Protein” tags. We propose to call this the LKG domain. A second less-well-conserved segment occurs between *Bombus* residues 75 and 106, with 16 sequence identities out of 32 residues. After these conserved regions, numerous sequence insertions and deletions occur across the three species with a long insertion of 23 residues starting from residue 145 found in *Lethocerus*. On this basis, the small, folded domain at the beginning of the density and conserved sequence at the N-terminus correspond.

Among the three reconstructions, the putative *Drosophila* myofilin has the smallest volume (3966 Å^3^). *Bombus* has about twice the volume of *Drosophila* (7429 Å^3^), and *Lethocerus* myofilin has the largest volume (10,540 Å^3^). The difference between the three myofilins occurs at the density downstream from the LKG domain (Figure 5B, labelled 1). Immediately following the LKG domain, *Bombus* myofilin seems to form a loop that extends sufficiently to reach the densities in the paramyosin core. Parts of this loop are present in *Lethocerus* or *Drosophila*, but most of the density there points towards the curved layers rather than towards the paramyosin core. *Bombus* myofilin then overlaps with the *Lethocerus* myofilin up to its intersection with the flightin density (Figure 5B, labelled 2). Following this intersection, the *Bombus* structure is disordered.

Another small density found in *Bombus* but unconnected to the main myofilin density (Figure 5B, labelled 3; Supplemental Video S3) superimposes *Lethocerus* myofilin at the downstream site. This small density accounts for about 20% of the *Bombus* myofilin volume and thus is assumed to be a part of the myofilin. This density may correspond to the second conserved region in the myofilin sequence. It contacts the same curved layer that the preceding segments contact and does not contact the paramyosin core. The missing segments of myofilin are apparently mostly disordered.

#### 2.4.3. Paramyosin

The *Bombus* thick filament has a high-density feature forming a central core that compares favorably to that of *Lethocerus* (Figure 5C). *Drosophila*, which has less paramyosin than *Lethocerus* and *Bombus*, effectively shows an empty core. Similar to *Lethocerus*, the *Bombus* paramyosin density showed up in the map, low pass filtered to 15 Å resolution, and presented as rod-shaped densities. However, the presumed *Bombus* paramyosin has a different density distribution compared with *Lethocerus*, with gaps just below the crown levels (dashed lines), whereas the comparable density in *Lethocerus* is continuous. This effect may occur because the paramyosin does not follow the myosin tail helical structure and is thus poorly represented in the reconstruction.

The distribution of these three non-myosin densities in the context of the thick filament shows that flightin and myofilin follow both the C4 symmetry and the helical symmetry of the myosin molecules (Figure 5D–F). Additionally, *Bombus* flightin, similar to that of *Lethocerus* and *Drosophila*, passes between myosin tails on the N-terminal side of the WYR motif, but it also passes myosin tails in a separate curved layer at the C-terminus. (Figure 5G, Supplemental Video S4). With its extended C-terminus observed here for the first time, flightin threads its way around and through five myosin curved layers, which may define its role in determining the thick filament helical symmetry and its mechanical properties [30]. The WYR motif of flightin has close contact with the Skip 3 accommodation region and on its C-terminal side has close contact with the curved layer at Skip 3.

The N-terminal LKG domain of myofilin that appears in common to all three species lies between adjacent curved layers with a following loop that, unique among these three species, extends into the paramyosin core of *Bombus*. In fact, both myofilin and flightin contact the paramyosin core (Figure 1C and Figure 5H,I; Supplemental Video S5), a more specific interaction cannot be identified due to the low resolution of paramyosin, but it is possible that three non-myosin proteins work together to stabilize the thick filament structure, determine its length, helical symmetry and alter its mechanical properties [30].

### 2.5. Protein Identification and Quantification by Mass Spectrometry

Even though no annotated genome is available for *Bombus ignitus*, bottom-up proteomics may be capable of detecting the presence and approximating the relative abundance of thick filament proteins using the *Bombus* datasets currently available. With this in mind, we digested six myofibril samples and one thick filament sample with trypsin, performed liquid chromatography mass spectrometry (MS), and identified and quantified peptide abundances in the resultant MS spectra. We reported the number of unique peptides that corresponded to known thick filament proteins and the relative abundance of each protein (Table 2). It is worth noting some of the limitations of this analysis. Many proteins may be highly conserved between *Bombus* species, but generate a limited number of peptides with the same exact mass as in *Bombus ignitus*, due to the position of the differences within the protein. This will limit protein sequence coverage and the overall confidence in the label-free approach to quantification, which relies on there being similarities in the ionization efficiency of top ionizing peptides from each protein.

The values reported in Table 2 are the relative abundance of each protein to a single myosin molecule, consisting of two myosin heavy chains and four myosin light chains. One molecule of flightin should exist for each molecule of myosin, based on the density observed with the thick filament. However, the stoichiometry determined (Table 2) is 25% of that value. Similarly, one molecule of myofilin should exist for each molecule of myosin, and it appears at 80% of that value. Five times as many myofilin peptides were observed in the analysis, consistent with its greater molecular mass. The greater accuracy of the latter value would result from there being less variance in the amino acid sequence of myofilin within the *Bombus* species.

No stretchin-klp-liked protein was observed in the *Bombus* thick filament image reconstruction or detected by MS, which helps exclude the possibility of its presence being responsible for the myosin head disorder, as was suggested for the disordered heads in *Drosophila* thick filaments [13]. However, phosphorylation of serine 62 and threonines 36 and 38 of the myosin RLC was detected. The peptides that contained phosphoserine 62 were ~4.5 times more abundant than the non-phosphorylated peptide. In contrast, the non-phosphorylated peptide that contained threonine 36 and 38 was ~3.5 times more abundant than those containing threonine 36 and threonines 36 and 38 phosphorylated. RLC phosphorylation is broadly believed to be a factor in breaking up the ordered heads of the interacting heads motif [31]. However, it is worth noting that an image reconstruction of *Drosophila* thick filaments with mutated RLC that could not be phosphorylated still showed disordered myosin heads similar to that of the wild type [13].

Unlike flightin and myofilin, which exist as monomers, paramyosin exists as a dimer, and this must be accounted for when comparing the density in the reconstruction and measured abundance. The measured ratio of a myosin molecule to each paramyosin molecule in *Bombus* was ~1:3.3. This is close to the proposed arrangement of paramyosin in *Lethocerus* with a 4-start helix and 725 Å axial translation [8]. We identified the *Bombus* species whose sequence for flightin and myofilin was most probably closest to that of *B. ignitus* based on the number of identical peptide masses observed in the MS experiments. If correct, this probably contributed to a more accurate model prediction using AlphaFold2 as well as improving the multiple sequence alignment.

### 2.6. Atomic Model Fitting

It is hard to construct an accurate atomic model of an unknown protein, de novo, at 6Å resolution, but with the advent of the molecular modeling program, AlphaFold2 [25], an atomic model of varying precision is attainable given a known protein sequence. Generally, AlphaFold2 works best on isolated domains [25,32], but given that it is easy to use, might shed some light on the atomic structure of some of these proteins, particularly the regions where the amino acid sequences are relatively well conserved. We used AlphaFold2 to predict the 3-D structure of flightin using the sequence from *Bombus vosnesenskii* (A0A6J3L7M6) and of myofilin using the sequence from *Bombus terrestris* (A0A6P3U2G4).

For flightin, AlphaFold2 predicted a reasonable atomic model for the WYR domain corresponding to residues 56 to 102. The reconstruction contained no density that could correspond to the N-terminal 40 residues which would be positioned outside of the thick filament backbone and likely disordered. The AlphaFold2 model fits the density well starting with the N-terminus of the WYR domain α-helix at residue 65, the following extended chain segment, the next short segment of α-helix, a short segment of the extended chain and the last α-helix. After the 3rd segment of α-helix, the C-terminal 25 residues, which resembled the tail density of *Bombus* flightin, did not fall within the observed density envelope. The AlphaFold2 model could be aligned well to the density from the 1st to the 3rd short segments of α-helix, which included the WYR motif. Everything else lay outside of the density envelope.

**Figure 6:**
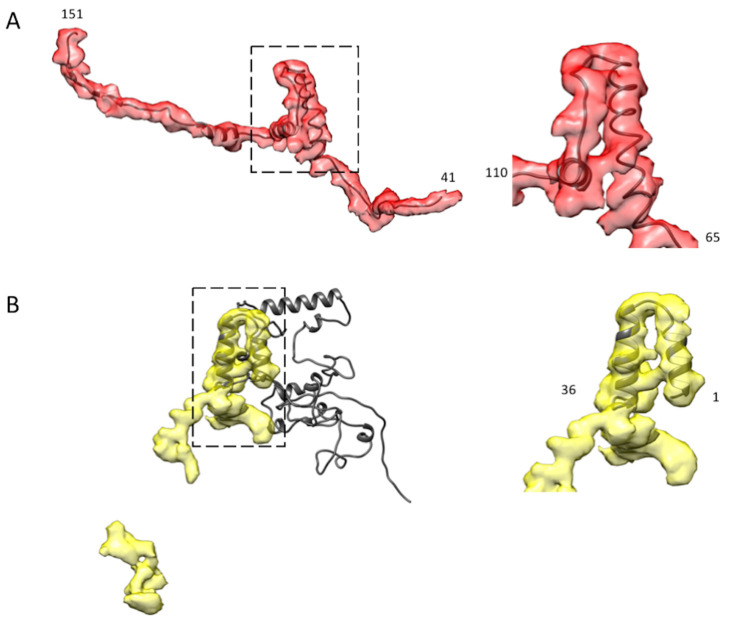
AlphaFold2 modeling of *Bombus* myofilin and flightin. (**A**) The AlphaFold2 atomic model of the flightin WYR motif fits well into the globular domain in a zoomed-in view (**right**) that roughly extends from residue 65 to residue 110, which is also the region of conserved sequence and similar reconstruction density in *Drosophila* and *Lethocerus*. The full atomic model starts from residue 41 to the residue 151 at the C-terminus (**left**). (**B**) The AlphaFold2 atomic model of myofilin only fits well at the N-terminal LKG domain up to residue 36 (**right**), which corresponds to the region of high sequence and density conservation in *Drosophila* and *Lethocerus*, while the rest of the AlphaFold2 model has poor reliability (**left**).

The model of the two ends of the flightin density was rebuilt de novo using Coot [33]. All the poorly aligned residues at the two ends were deleted, and then a polyalanine chain was fitted into the density, after which the polyalanine was mutated to the correct flightin sequence. The model was refined using several rounds of real-space-refinement in Phenix [34] until the model fit the density with an absolute value of Ramachandran Z-score under 3 (Figure 6A, Supplemental Video S6). The final atomic model was evaluated by the wwPDB validation service.

The AlphaFold2 model of myofilin was quite complex and compact, with only the N-terminal LKG domain (about 36 amino acids) fitting the density well (Figure 6B, Supplemental Video S7). The LKG domain happens to be the domain whose sequence and shape are shared by *Bombus*, *Lethocerus*, and *Drosophila*. However, the rest of the model is a total puzzle, with low reliability. Only about 20% of the AlphaFold2 myofilin atomic model fits the density with any reasonable reliability. We did not attempt to build an atomic model of myofilin because of the relatively poor coverage of the AlphaFold2 atomic model with the observed density and the significant gap between the two densities that we believe are from myofilin.

## 3. Discussion

Among all striated muscles, the thin filament structure is more conserved regarding protein composition and structure than the thick filament. While thick filaments from vertebrate striated muscle are quite similar, thick filaments from invertebrate striated muscle are quite variable in length, rotational symmetry, and protein composition. The *Bombus ignitus* flight muscle thick filament structure contains features previously reported for *Lethocerus* and *Drosophila* structures but has differences as well. Thick filament structures have now been reported for three of the four insect orders that have evolved asynchronous, indirect flight muscles. A comparison among the three thick filament structures might reveal the fundamental characteristic of asynchronous flight muscles and reveal characteristics of striated muscle thick filaments in general.

### 3.1. The General Features of Asynchronous Flight Muscle Thick Filaments 

General features of thick filaments include their length, diameter, axial rise, helical angle, and rotational symmetry. Of these, the latter three parameters are nearly identical for the three thick filaments (Table 1). The axial rise of 145 Å was determined independently from X-ray fiber diffraction [35,36,37,38,39]. If there is a difference in axial rise, detecting it will require a very precise determination.

A slight difference in filament diameter is seen with *Drosophila* having a slightly smaller diameter than either *Lethocerus* or *Bombus*, whose diameters are nearly identical. All three thick filaments consist of an outer annulus of myosin tails and a central core, which is occupied by paramyosin, which we refer to as the paramyosin core. The paramyosin content parallels the amount of density seen in the paramyosin core. The paramyosin to myosin ratio is comparatively high for *Lethocerus* [8,40,41] and *Bombus* (Table 2), and significantly lower for *Drosophila* [13,42]; the *Lethocerus* and *Bombus* reconstructions had rod-shaped densities in their paramyosin core, while for *Drosophila*, the central core was empty. The reconstruction process enforces the myosin helical symmetry so that only those features that follow the myosin symmetry are well represented. Paramyosin, with a length slightly longer than 8 × 145 Å, forms structures characterized by an axial repeat of 5 × 145 Å. The larger diameters are apparently enforced by high paramyosin occupancy in the core.

### 3.2. Nonmyosin Densities

We observe four non-myosin densities in the three species of insect studied at a subnanometer resolution: stretchin-klp, paramyosin, flightin, and myofilin. We observed stretchin-klp only in *Drosophila* and only on the surface of the thick filament [13,14]. The thick filament surfaces of *Bombus* and *Lethocerus* were free of surface proteins. All three showed parts of the flightin N-terminus to varying extents. We do not observe paramyosin in *Drosophila* because there is too little of it to present a feature when the helical symmetry of the myosin is enforced. This seems to be a feature of flies in general, as transverse sections through various species show hollow filaments [43]. In *Lethocerus* and *Bombus*, paramyosin presents as uncoordinated rod-shaped densities in the core of the filament. Paramyosin, whose structure is a coiled-coil of slightly more than eight crowns in length, is not represented accurately in the two reconstructions because it most likely follows a symmetry different from the myosin. The other two proteins, flightin and myofilin, are observed in all three, the comparison of which helps to define elements of their structure in more detail than previously possible.

Flightin is essential for maintaining insect flight muscle structure and performance; when flightin is eliminated in *Drosophila* flight muscle myofibrils, defective myofilaments are formed and flight is severely impaired [44]. Flightin in one form or another has been found in 69 species, not all of which can fly [28]. Flightin sequences vary in length but have a strictly conserved region in the middle, dubbed the WYR motif that spans over ~50 amino acids that are critical for normal flightin function [28]. More significantly, the WYR motif is said to share a binding site on the myosin coiled-coil with vertebrate myosin binding protein C [28].

Here with *Bombus*, we observe for the first time what appears to be the complete density of the part of flightin embedded within the thick filament from the C-terminus deep within the backbone to residue 41, the first part of flightin stabilized by contact with the proximal S2. The shape of this density strongly supports its assignment to flightin due to the presence of a folded domain in the middle in the predicted position of the “WYR” motif. The WYR motif density contacts two adjacent curved layers as if to stitch them together. On the WYR motif N-terminal side, flightin passes between two myosin tails in the vicinity of the Skip 3 accommodation region of one tail. The C-terminus of flightin contacts the Skip 3 region of one myosin tail of the last curved layer it spans over. Skip 3 is believed to stabilize the coiled-coil structure [45]. According to the flightin sequence alignments, conservation is poor on either side of the WYR motif (Figure 4A) in different insects adapted to different environments. The N-terminal region (residue 1–40) is disordered in our reconstruction but has a relatively conserved pattern that starts with an acidic residue-rich region followed by an alanine and proline-rich region [46], which favors interaction with the solvent and neighboring actin filament, and, for this reason, the flightin N-terminus is not resolved in our reconstruction. Our reconstruction does suggest an interaction between flightin and the proximal S2 starting at about flightin residue 41.

Our more complete density of flightin resolves the identity of the so-called “blue” density first seen in *Lethocerus* thick filaments [8] and later in *Drosophila* [13]. In *Lethocerus* the “blue” density superimposes the *Bombus* C-terminal end of flightin (Figure 5A, Supplemental Video S2), arguing its assignment as the flightin C-terminus. The C-terminus of flightin is more conserved than the intervening sequences following the WYR motif which also vary in length. Multiple sequence alignment has shown many conserved residues at the C-terminus with high similarities over a larger species range (Figure 4A), which may explain the presence of the “blue” density in all three species. The intervening density crosses over *Bombus* myofilin but does not seem to contact the underlying myosin tails, which may account for its disappearance in *Lethocerus* and *Drosophila*. The C-terminus (a.k.a. “blue” density) is sandwiched between a pair of curved layers but does not come close to the paramyosin core and thus seems not to be involved directly in binding paramyosin. However, the way the flightin C-terminus passes over a curved layer to contact two other curved layers suggests an important role in establishing the thick filament structure because it may modify or define the separation between curved layers near the paramyosin core.

Genetic studies on *Drosophila* indicate that truncation of the C-terminal 44 residues of flightin produces filaments of variable length thereby disrupting the highly regular flight muscle sarcomere needed for flight [27,47]. Flightin nulls disrupt both thick filament length as well as the highly regular sarcomeres. The exact mechanism of invertebrate thick filament length determination is unknown but both flightin, in *Drosophila*, and paramyosin, in *C. elegans* [48], are involved. Flightin from the WYR domain to the C-terminus is located on the inside surface of the myosin annulus. The WYR domain itself is the only part of flightin that extends sufficiently inward from the myosin annulus to contact paramyosin. The WYR motif serves an important function for association with myosin because truncation of either the N- or C-terminal residues does not affect flightin incorporation into the thick filament [27].

For myofilin, previous sequence alignments among *Anopheles gambiae*, *Drosophila* and *Lethocerus* indicated a highly conserved sequence on the N-terminal domain and poor conservation near the C-terminus [49]. The same pattern is observed here (Figure 4B). All three of our reconstructions have shown a globular protein density in the same location at one end of an extended density that is highly variable among the three species. The highly conserved sequence at the N-terminus we suggest corresponds to the overlapping folded domain in *Drosophila*, *Lethocerus*, and *Bombus*. After converting the volume size to a sequence length (0.8 Da/Å^3^; 110 Da/amino acid), the folded domain is about 38 amino acids, which is similar to their common sequence length (about 35 residues) and close to the length of the atomic model fit into the density map (36 residues), all of which support the assignment of the folded domain as the myofilin N-terminus. The sequence length of the various species of myofilin is much larger than the myofilin volume in our structure, indicating that there are more densities yet to be resolved or that are disordered. *Lethocerus* has the longest and most complete myofilin density among these three. Unlike the continuous myofilin density in *Lethocerus*, *Bombus* myofilin consists of two unconnected pieces that overlap the continuous myofilin density of *Lethocerus*. In *Bombus*, the extended flightin C-terminal domain contacts myofilin downstream of the LKG domain, so an interaction between these two non-myosin densities should be expected.

Unlike flightin, whose extended length contacts up to five curved layers in each asymmetric unit, myofilin interactions that have been observed are largely confined to a single curved layer. The LKG domain contacts a pair of curved layers, but the rest of the myofilin densities in the three species seem to be confined to a single curved layer. As such, myofilin may function to modify the structure of the curved layer, which could affect features such as the helical pitch. Flightin on the other hand, contacts many curved layers and thus might function more in the role of determining the relative positioning of the curved layers. The LKG domain of myofilin, similar to the WYR motif of flightin, extends far enough into the paramyosin core to contact paramyosin. This contact may be important for determining the filament length.

Paramyosin has been found overwhelmingly in invertebrates [50] and is present in both their smooth muscle and striated muscles [40,51]. (An unpublished search by us found paramyosin in all invertebrates.) The amount of paramyosin relative to myosin differs considerably in different muscles and thus may affect the diameter of the thick filament, its rotational symmetry, its mechanical properties, and, in concert with other proteins, its length [24]. Paramyosin aggregates of various types have an axial spacing of ~725 Å [41,52,53,54,55]. All invertebrate thick filaments that have been examined by X-ray fiber diffraction to determine their axial repeat were found to have a spacing of 145 Å [38,39,56,57,58]. The lowest rotational symmetry so far found for invertebrate thick filaments is 4-fold [59,60,61,62,63,64,65,66,67]. The loss of paramyosin in vertebrates may be responsible for the 3-fold symmetry, which is found only in vertebrates, and the average 143 Å axial spacing of relaxed vertebrate thick filaments. Activated vertebrate thick filaments have an axial spacing of 145 Å, the same as the relaxed invertebrate thick filaments [68,69,70]. The protein titin determines the length of vertebrate thick filaments which, in so far as it is known, all have a length of 1.6 μm [71,72,73,74,75]. Arguably, loss of paramyosin allowed vertebrate thick filaments to evolve a novel mechanism for thick filament activation and relaxation, the details of which are still unknown. 

### 3.3. The Disordered Myosin Heads

An ordered IHM is observed in *Lethocerus* and many other striated muscles but is absent in *Bombus* and was not observed for *Drosophila* [13]. Whether the IHM forms in *Drosophila* thick filaments but is disordered is an open question. If the IHM is formed in relaxed *Drosophila* thick filaments, it appears unable to bind the thick filament backbone. Assuming that the docking of the IHM against the thick filament backbone would be similar in *Drosophila* and *Lethocerus*, all other things being equal, the binding site used by *Lethocerus* is blocked in *Drosophila* due to the presence of the protein stretchin-klp [13]. Moreover, IHM formation in purified flight muscle myosin from *Drosophila* was found to occur but was less stable than its embryonic myosin counterpart [10].

Three factors are known to affect the formation of the IHM, temperature in vertebrate striated muscle [76,77,78], mutation of key residues involved in stabilizing the structure, which in humans leads to various cardiomyopathies [11] and RLC phosphorylation [31]. No temperature effect on the ordering of myosin heads in an insect flight muscle, either synchronous or asynchronous, has been reported to our knowledge. The cryoEM specimens of *Lethocerus* flight muscle were frozen at 4 °C; all had ordered myosin heads [8,21].

Myosin head disorder might be due to changes to the residues at the interface between the thick filament backbone and the Free Head such as occurs with the Free Head in *Lethocerus*. At the moment, a high-resolution structure of the *Lethocerus* thick filament with the Free Head is not available nor is the sequence of *Bombus ignitus* flight muscle myosin. The unique orientation of the Skip 1 accommodation region in *Bombus* (Figure 2A) might be causative. The accommodation region following a skip residue is the segment of coiled-coil that untwists, enabling the individual α-helices to unwind sufficiently to accommodate the added amino acid residue. The Skip 1 accommodation region is positioned between the crown levels, so a change in its orientation might reposition key residues that interact with the Free Head.

In the case of *Drosophila*, RLC phosphorylation as a cause of myosin head disorder can be ruled out by the fact that an ordered IHM did not form in a strain in which the phosphorylation sites had been mutated out [13]. However, the IHM might have formed but been disordered. The mass spectrometry analysis in the present work indicates that phosphorylation had occurred at several sites on the *Bombus* RLC which might be responsible for the disordering of the myosin heads. However, only about 25% of the RLCs were phosphorylated (Table 2). The explanation is complicated if similarities with the *Lethocerus* thick filament are considered. In *Lethocerus*, the Blocked Head is already poorly ordered in the absence of phosphorylation and, even if fully phosphorylated, would not necessarily disorder the Free Heads which can bind the thick filament backbone independent of the state of the Blocked Head.

The disordered heads of *Bombus* thick filaments raise a couple of issues regarding how stretch activation might work as well as the control of heat production in the muscle.

### 3.4. Effect of Disordered Myosin Heads on Stretch Activation

Asynchronous flight muscle is characterized by an extremely well-ordered myofilament lattice, and contractions that take place following a stretch, i.e., stretch activation [79]. In *Lethocerus*, stretch activation has been shown to occur at submaximal calcium concentrations [80]. Many features of the flight muscle lattice have been shown to affect stretch activation including troponin-C isoforms [81], flightin [82,83], the N-terminal extension on the RLC [84], extensions on tropomyosin and troponin [85], and troponin bridges formed from myosin heads [38]. Another stretch activation model proposes that stretch produces enhanced alignment of myosin head origins with actin targets [59]. Supporting this idea is the observation that a stretch of relaxed *Lethocerus* myofibrils also enhances the alignment of myosin heads with actin targets through the unwinding of right-handed helical tracks on the thick filament [38].

A mechanism that relies simply on the alignment of myosin head origins and actin targets may be insufficient by itself to explain stretch activation because the thin filaments are not fully activated and the myosin heads in the pre-stretched muscle may be folded into an IHM which seems likely to occur in *Lethocerus*. The stretch itself must fully activate at least a fraction of myosin binding sites on actin and disrupt a fraction of the IHMs to enable myosin heads to bind actin target zones. As proposed [38], so-called Tn bridges may act to push (or pull) Tm into the fully activated (or open) position within the target zones to initiate force production.

Stretch activation in insect flight muscle also requires a shortening deactivation because the calcium concentration stays relatively steady. In the *Lethocerus* flight muscle, the muscle only shortens ~3% of the sarcomere length or 387 Å [86]. It has been argued that IHM formation could occur between muscle contractions in both *Lethocerus* and *Drosophila* (see the Supplemental Material in references [8,87]) but its rate of formation in any myosin II, as far as we know, has not been measured directly. In *Lethocerus*, where the IHM is ordered against the filament backbone, this mechanism seems at least plausible. However, in *Drosophila* and *Bombus*, a disordered IHM may not even form. The utility of a disordered IHM in possibly accelerating muscle relaxation is uncertain. A disordered IHM buries the actin-binding interface of the Blocked Head against the Free Head thereby reducing the number of myosin heads capable of binding actin by a factor of two and may also place the Free Head in an unfavorable position for binding actin. Potentially, because the paired heads must move about a pair of α-helices instead of a single α-helix if both heads were independent, a disordered IHM might be less mobile, thus accelerating relaxation provided it can form fast enough for the high wing beat frequencies found in *Drosophila* and *Bombus*.

If the IHM or some related sequestration of myosin heads does not form between wing beats, what other mechanism could contribute to shortening deactivation? An early proposal offered as an explanation for stretch activation suggested that at least in *Lethocerus*, the highly regular arrangement of thick and thin filaments created a periodicity to the opportunities for myosin heads to strongly bind actin filaments and with it the possibility that shortening of the muscle would reduce those opportunities [59].

### 3.5. The Disordered Myosin Heads Effect on Muscle Temperature

The super-relaxed state is defined as one with low ATP consumption [88]; the most likely structure of myosin heads that would contribute to this state is the IHM due to its ability to sequester both myosin heads from actin binding. The IHM not only sequesters myosin heads from interacting with actin, but also reduces the myosin basal ATPase rate, which does not require actin [88]. By minimizing ATP consumption, the super relaxed state minimizes heat production in the muscle [89]. The IHM has been observed in many myosin II isoforms isolated from different species of multicellular organisms, which suggested universality [10,90] and has been observed in relaxed thick filaments isolated from many striated muscles [3,4,5,6] and one unusual smooth muscle [2]. Some RLC phosphorylation, ~5%, is necessary to activate some myosin heads and transfer the muscle into a disordered-relaxed state [88,91].

In *Lethocerus*, flight muscle force-producing myosin heads only occur on four actin subunits, two on each long-pitch helical strand, midway between troponin complexes on each thin filament [92,93]. The axial periodicity of these target zones is the same as the troponin spacing, 387 Å. The *Lethocerus* axial period of 1160 Å (8 × 145 Å or 3 × 387 Å) contains 18 target zones on the six thin filaments surrounding each myosin filament, and 64 myosin heads that could potentially bind to generate force. This likely means that, during contraction, no fewer than 18/64 (28%) and no more than 36/64 (56%) myosin heads can be generating force on each thick filament during each contraction. The remainder, if not in the IHM, is likely cleaving ATP at the basal level. The basal ATPase rate in glycerinated *Lethocerus* flight muscle fibers has been measured and is between 1/9th and 1/3rd of the actin-activated ATPase rate during active contraction [86]. Since these heads are doing no work, the free energy of ATP cleavage is likely given off as heat, warming the thorax and contributing to keeping the thorax above the 40 °C needed to maintain flight. If this occurs in *Bombus* all the time, the basal ATPase rate is contributing in a small way to warming the thorax when not flying and maintaining the thorax above 40 °C during flight.

The lack of an IHM structure and its potential implication for achieving super relaxation in *Bombus* and *Drosophila* may relate to their lifestyle. As described in his book [94], Heinrich argues that flying insects, both those whose flight muscles are synchronous and asynchronous, must elevate their thorax to temperatures of 40 °C or more in order to fly, presumably because the elevated temperature leads to higher myosin ATPase activity to support faster contraction frequencies. The thorax of flying insects is almost all muscle, as the various organs to sustain life are located in the abdomen where they need not be subjected to the elevated temperature of the thorax when flying. *Lethocerus indicus*, order Hemiptera, is a giant water bug and spends most of its time underwater, where its wings are useless. It flies mainly to move from one water source to another. A super relaxed state makes perfect sense for the giant waterbug.

Although making a generalization based on just three observations is risky, we think the following is worth considering. Four insect orders have evolved asynchronous flight muscles: *Drosophila melanogaster*, order Diptera, and *Bombus ignitus*, order Hymenoptera, are two species of insect that are flying around all the time. Bumble bees, spend little time on a particular flower and move after a few seconds. To fly at a moment’s notice requires maintaining thoracic temperatures at 40 °C even when not flying, which, according to Heinrich, is done by “shivering”. *Bombus* sp. can be found in extremely cold environments where they must generate sufficient heat from their thorax, to warm their larvae. Thus, their flight muscles quite possibly never or rarely go into a super-relaxed state. Development of the IHM may not have occurred in these species because it was never useful, which tempts a prediction.

Insects of the Coleoptera order, which includes beetles, may have developed a super relaxed myosin head conformation (an ordered IHM) because they spend a lot of their time not flying. Dung beetles, for example, spend much of their time rolling around balls of dung. Pine borers spend much of their time tunneling through wood. Japanese beetles remain parked on plants while eating away at them. It will be interesting to see how well- ordered are the myosin heads of beetle asynchronous flight muscles.

## 4. Materials and Methods

### 4.1. Thick Filament Preparation

Dissected thoraces from bumble bees of the species *Bombus ignitus* collected in the wild in Japan and provided by Dr. Hiroyuki Iwamoto. They were stored at −80 °C in a relaxing buffer containing 70% glycerol (80 mM K-propionate, 20 mM imidazole, 10 mM EGTA, 4 mM Na-ATP, 5 mM MgCl_2_, 50 μg/mL leupeptin, pH 7.2). For the thick filament preparation, about one and a half thoraces of *Bombus* flight muscle were used. They were rinsed with the relaxing buffer (150 mM NaCl, 5 mM Mg acetate, 5 mM NaATP, 5 mM EGTA, 1 mM DTT, 20 mM MOPS, pH 7.0) plus 1% protease inhibitor cocktail (Product brand: Sigma, Product number: 2714), then homogenized in a 1 mL ground glass homogenizer with 1 mL relaxing buffer. Myofibrils were obtained by centrifugation at 7000× *g* with turning of the centrifuge tube 3 times for 3 min each time, followed by 8000× *g* with one turn (1 time, 3 min). The pellet was resuspended in 0.3 mL relaxing buffer + 0.5% Triton X-100 to dissolve remnants of the cell membrane, then centrifuged again at 8000× *g* with four turns of the centrifuge for 3 min each to wash out the Triton. Another Triton treatment was performed, after which myofibrils were left in the relaxing buffer overnight.

The next day the myofibrils were centrifuged at 8000× *g* with turns of the centrifuge tube (4 times, 3 min each) and then resuspended in 0.12 mL calpain buffer (10 mM MOPS, 150 mM NaCl, 5 mM Mg acetate, 5 mM ATP, 5 mM EGTA, 3 mM DTT, 5.2 mM CaCl_2_, pH 6.8) plus 3 μL calpain (1.92 mg/mL) at room temperature (Athens Research and Technology, Athens, GA 30601, USA) to digest the Z-disks. After one hour, digestion was stopped by adding 0.24 mL Stop buffer (20 mM MOPS, 150 mM NaCl, 5 mM Mg acetate, 15 mM EGTA, pH 6.8). Digested myofibrils were then separated by centrifuging at 8000× *g*, again with four turns of 3 min each, with the supernatant discarded. Myofibrils were sheared by pulling the suspension 12 times through a 1 mL syringe with a 26 G needle in 35 μL Shear buffer (20 mM MOPS, 20 mM Na_2_HPO_4_, 100 mM NaCl, 5 mM Mg acetate, 5 mM ATP, 5 mM EGTA. 5 mM DTT, pH 6.8). Large solids were removed by centrifuging at 3500× *g* with 2 turns (2 turns, 2 min each). Then 20 μg of Ca^2+^-insensitive, His-tagged gelsolin was added to remove the actin filaments [8]. The preparation was evaluated and optimized with respect to the quality of preservation and thick filament concentration using uranyl acetate and the negative stain electron microscopy on a Philips CM120 electron microscope (Philips Electronic Instruments, Mahwan, NJ, USA).

### 4.2. Grid Preparation

About 5 μL of the specimen was applied on each of the plasma-cleaned 2/1 copper Quantifoil grid and back-blotted [8,13,95]. Plunge-freezing was performed in liquid ethane using a home-built plunge-freeze device installed in a 4° cold room.

### 4.3. Data Collection

Data were collected on a Titan Krios transmission electron microscope (Thermo Fisher, Hillsboro, OR, USA) operated at 300 keV. Images were recorded on a Gatan (Gatan, Inc., Pleasanton, CA, USA) K3 camera operated in counting mode. Each movie has 75 frames, and the defocus range is from −0.8 to −2.5 μm, with a total dose of 60 *e*^−^/Å^2^ and pixel size of 1.1 Å.

### 4.4. Data Analysis

After motion correction and CTF estimation using Relion [19], 11,708 thick filaments were picked manually by a selection of two points, as shown in the Workflow (Supplemental Figure S2). Using the beginning and ending points of the filament, segments five crowns in length, stepped along the filament successively by one crown, were selected. Approximately 120,000 filament segments (particles) were cut out and stored in 768 × 768-pixel boxes using Relion [19]. Segments were polished by 2D classification using cisTEM [15], which left ~1,100,000 segments for reconstruction. We tried different alignment references and rotational symmetries; the best one proved to be a *Lethocerus* reconstruction enforced with C4 symmetry and low pass filtered to 60 Å resolution. The local resolution was estimated using Local Monores [96], the map was sharpened by Local Deblur [97], and both programs were installed in Scipion [98]. The mask for sharpening, which is the key to improving the sharpening quality, was made by Relion. Relion was also used to determine the helical rise and twist, after which the helical symmetry was imposed to smooth the surface and also to extend the length of the reconstructed thick filament to 12 crowns. Map segmentation was carried out using Chimera [20,99].

### 4.5. Protein Identification and Quantification by Mass Spectrometry

To identify and quantify thick filament protein abundances, six myofibril samples and one thick filament sample were digested with trypsin and analyzed by liquid chromatography mass spectrometry (LCMS) as previously described [100]. Briefly, the individual samples were solubilized in RapiGest SF Surfactant (Waters Corporation, Milford, MA, USA), reduced, alkylated, and digested with trypsin (Promega Corp., Madison, WI, USA) to produce peptides. The peptides were separated by ultra-high pressure liquid chromatography and directly infused into Q Exactive Hybrid Quadrupole-Orbitrap mass spectrometer (Thermo Scientific, Waltham, MA, USA). Peptides were identified from the resultant mass spectra by searching against a custom proteome containing all protein sequences from *Bombus* (accessed from UniProt 16 September 2021) using SEQUEST, in the Proteome Discoverer, version 2.2 (PD 2.2) software package (Thermo Fisher Scientific, Waltham, MA, USA). The LC peak areas within each sample were quantified using the Proteome Discoverer 2.2 (PD 2.2) software package with the Minora Feature Detector enabled (Thermo Fisher Scientific, Waltham, MA, USA). The LC peak areas were normalized using the abundance of the top three peptides that corresponded to myosin heavy chain from *Bombus terrestris*. Next, we estimated the number of molecules of each thick filament protein, relative to a single myosin molecule, from the average abundance of the top three peptides of each protein. These values were multiplied by a factor of two to account for each myosin molecule being a dimer of two heavy chains, except for paramyosin, which is also a dimer. The average relative abundance of each thick filament protein and the standard deviation of the measurements from the seven samples were reported. Raw data can be found in Supplementary Table S1.

### 4.6. Protein Sequence Comparison

Multiple sequence alignment was performed on flightin and myofilin from three insect species using Clustal W. The closest *Bombus* species to *B. ignitus* based on the mass spectrometry experiments were used to obtain the accession codes from Uniprot (accessed 25 October 2021). For flightin, *Drosophila* (P35554), for *Lethocerus* (Q5GMQ5) and for *Bombus* (A0A6J3L7M6) were aligned (Figure 4A). For the myofilin, sequences from three species were compared in the same order: *Drosophila* (Q8I062), *Lethocerus* (Q70VH9) and *Bombus* (A0A6P3U2G4) (Figure 4B).

## Figures and Tables

**Table 1:** The comparison of the helical parameters between three species.

	*Lethocerus*	*Drosophila*	*Bombus*
Helical rise (Å)	147.038	148.826	147.533
Helical twist (˚)	33.98	33.86	34.05

**Table 2:** Thick filament proteins and relative molar abundances quantified by mass spectrometry.

Thick Filament Protein ^§^	Accession Number for Peptides	Species	# Myosin Molecules per Molecule ± SD (n = 7)	Number of Peptides Detected	Phosphorylation Site
Myosin heavy chain	A0A6P5HR22; A0A6P3TVX4	*B. terrestris*	0.50 ± 0.00	189	
Myosin essential light chain	A0A6P3UB85	*B. terrestris*	0.59 ± 0.12	12	
Myosin regulatory light chain	A0A6P8MQT; A0A6P3UC24	*B. bifarius;* *B. terrestris;*	1.45 ± 0.46	17	S 62, T 36, T 36 + T 38
Flightin	A0A6J3L7M6	*B. vosnesenskii*	4.16 ± 0.43	5	
Myofilin	A0A6P3U2G4	*B. terrestris*	1.25 ± 0.07	28	S 140
Paramyosin	A0A6P3TYQ7	*B. terrestris*	3.29 ± 0.38	85	S 872
Mini-paramyosin	A0A6P5HKR2	*B. terrestris*	37.93 ± 11.75	19	
Twitchin/Projectin	A0A6P8MB74 A0A6P5I285	*B. bifarius;* *B. terrestris*	20.32 ± 8.60	349	
Obscurin	A0A6P3DZQ5 A0A6P3D8J7 A0A6P3UZW6	*B. impatiens* *B. terrestris;*	47.98 ± 29.44	97	
Titin	A0A6J3LFW1 A0A6P5HR09	*B. vosnesenskii;* *B. terrestris*	33.72 ± 17.88	179	S 4918
Titin	A0A6P3URE2 A0A6P3U365 A0A6J3KGZ5	*B. impatiens;* *B. terrestris;* *B. vosnesenskii*	10.75 ± 13.44	68	
Titin isoform X1	A0A6P3TXU4	*B. terrestris*	13.66 ± 6.61	21	

^§^ Protein name assigned to the accession code by Uniprot.

## Data Availability

The full reconstruction of *Bombus* thick filament as well as the segmented flightin density have been deposited in EMDB under accession code EMD-28208. The atomic model of *Bombus* flightin is deposited in the PDB under the entry 8EW5.

## Supplementary Materials

The following supporting information can be downloaded at: https://www.mdpi.com/article/10.3390/ijms24010377/s1.
