# Supplementary material for: Structure of the Flight Muscle Thick Filament from the Bumble Bee, Bombus ignitus, at 6 Å Resolution
Source: Int J Mol Sci. 2022 Dec 26;24(1):377. doi: 10.3390/ijms24010377 (PMC9820631; doi:10.3390/ijms24010377)
Supplement: Supplementary file 1 [file ijms-24-00377-s001.zip › Descriptions of Supplementary Materials.pdf]

Descriptions of Supplementary Materials

The zip file contains the following:

A pdf containing
- Supplementary Figure S1, which shows the resolution determination
- Supplementary Figure S2, which provides a flow chart of the data analysis
- Legends for the 7 videos provided

An Excel file containing the raw mass spectrometry data

7 video files named:
- Li et al,2022,Video_S1.mp4
- Li et al,2022,Video_S2.mp4
- Li et al,2022,Video_S3.mp4
- Li et al,2022,Video_S4.mp4
- Li et al,2022,Video_S5.mp4
- Li et al,2022,Video_S6.mp4
- Li et al,2022,Video_S7.mp4
