# Supplementary material for: Structure of the Flight Muscle Thick Filament from the Bumble Bee, Bombus ignitus, at 6 Å Resolution
Source: Int J Mol Sci. 2022 Dec 26;24(1):377. doi: 10.3390/ijms24010377 (PMC9820631; doi:10.3390/ijms24010377)
Supplement: Supplementary file 1 [file ijms-24-00377-s001.zip › Li et al, 2022, Supplementary_materials,v3.pdf]

Structure of the flight muscle thick filament from the Bumble Bee, *Bombus ignitus*, at 6 Å Resolution

Jiawei Li, Hamidreza Rahmani, Fatemeh Abbasi Yeganeh, Hosna Rastegarpouyani, Dianne W. Taylor, Neil B. Wood, Michael J Previs, Hiroyuki Iwamoto and Kenneth A. Taylor

Legends to Supplemental Figure File

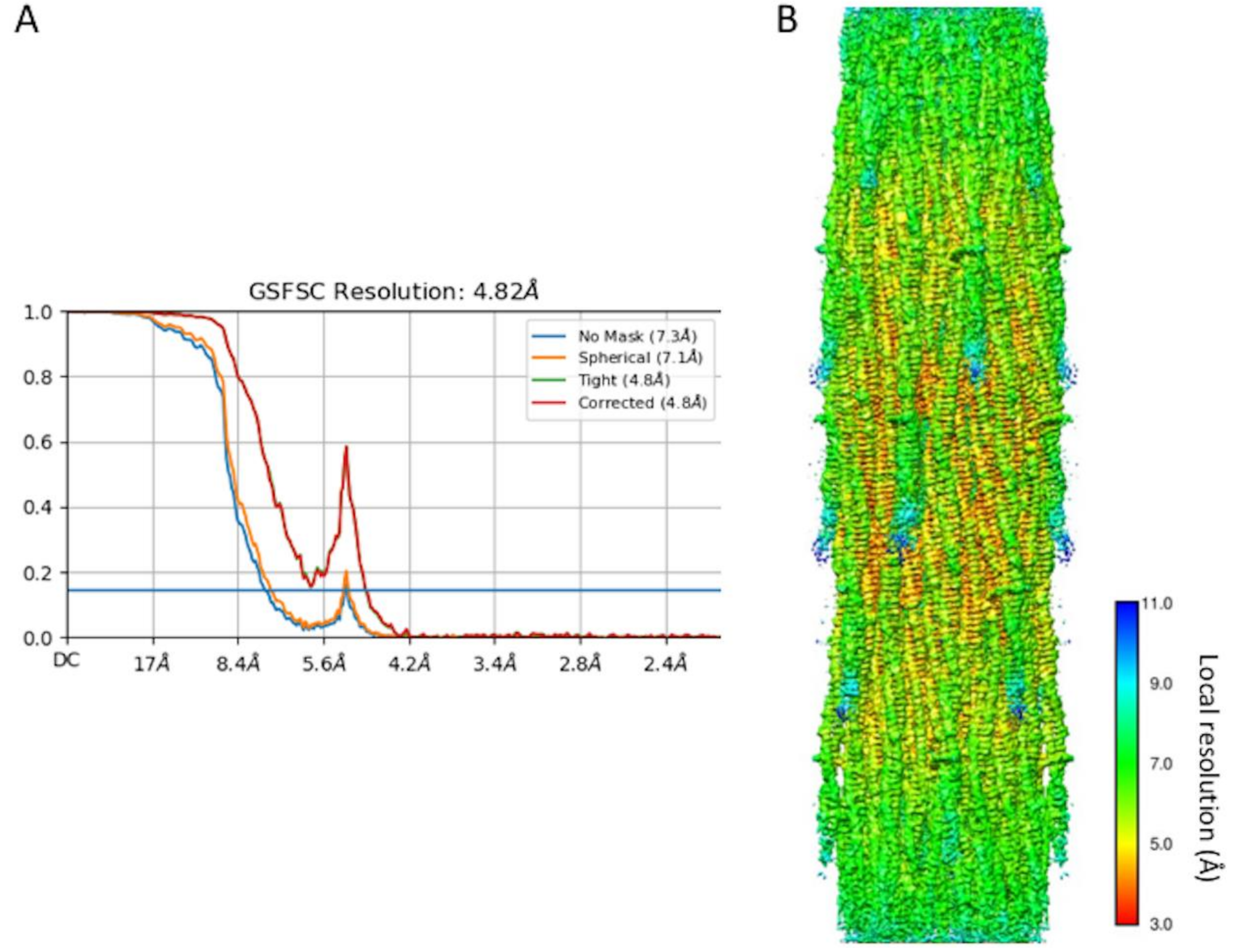

Figure S1. Validation of *Bombus* thick filament resolution. (A) The FSC curved calculated by CryoSPARC [16] to compare with the cisTEM, the resolution is still over-estimated as 4.82 Å, as no high-resolution features like side chains is visible in the map. The peak in the FSC curve at 5.17 Å resolution arises from the high α-helix content of the structure. (B) Using Local resolution estimation in CryoSPARC [16], a local resolution map was calculated and colored as a heat map. The resolution of myosin tail density is around 6 Å to 7 Å, which is comparable to the myosin thick filament map of *Lethocerus* [8], the resolution gradually goes down to 11 Å when it nears the proximal S2 region.

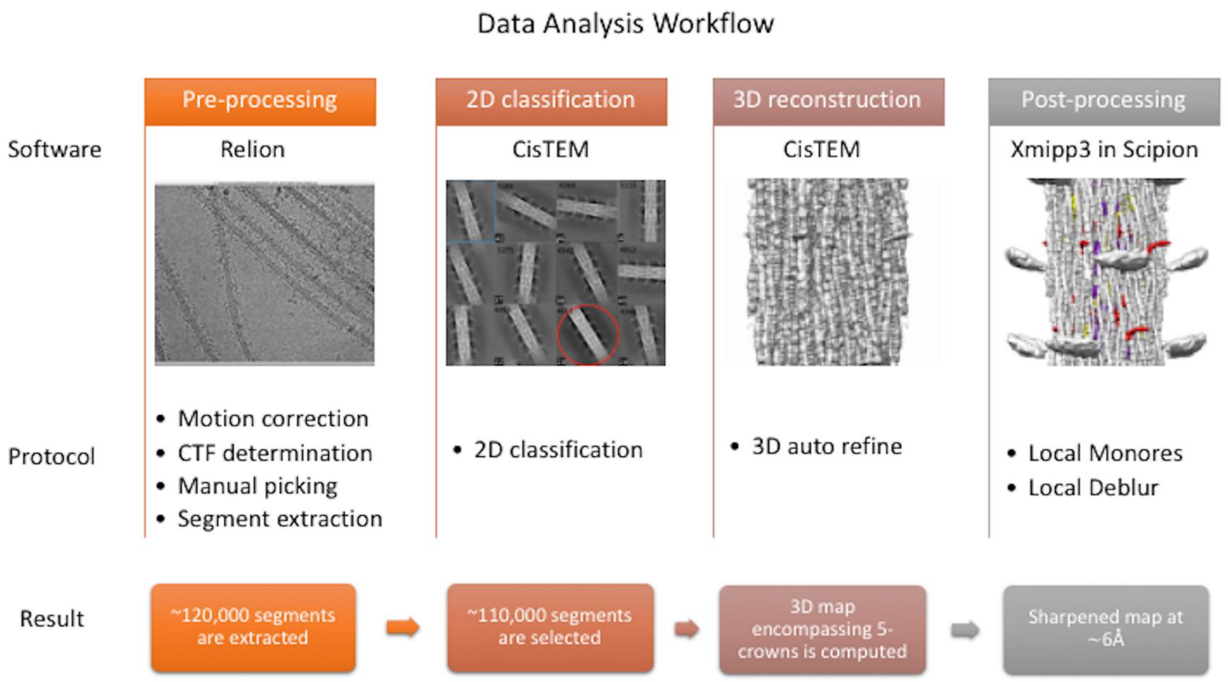

Figure S2. Data analysis work flow.

Legends to Supplemental Video Files

Video S1. Curved layer comparison between *Bombus* and *Lethocerus*. Superimposing the *Lethocerus* curved layer (white mesh) with the *Bombus* curved layer (blue solid) illustrates the relationship of the floating density, presumably representing the average density of mobile myosin heads with the proximal S2. At the beginning, the alignment of curved layers shows how the *Lethocerus* Proximal S2 is bent azimuthally by 17°. The trajectory of the Proximal S2 from *Bombus* S2 is predominately axial pointing straight down to the floating density without tilt compared to the *Lethocerus* S2. The cardiac S2 when fitted to follow the *Bombus* Proximal S2 direction, appears to connect to the floating density.

Video S2. *Bombus* flightin. *Bombus* flightin (red solid surface) shows the largest continuous density so far observed that superimposes well on the flightin density found *Lethocerus* (red mesh) also the previously unidentified “blue” density (blue mesh) from a recent, *Lethocerus* reconstruction. We interpret the region where the blue mesh and red solid surface overlap to represent the C-terminus of flightin, where the sequence conservation is higher than the intervening region between the WYR motif and the C-terminus.

Video S3. *Bombus* myofilin. The segmented density of *Bombus* myofilin (yellow, solid) occurred in two separate pieces, both of which overlap with *Lethocerus* myofilin (yellow, mesh). The N-terminal domain at the top corresponds to the LKG domain of myofilin, which is the most conserved region of the sequence. The second *Bombus* domain overlaps partially with

Lethocerus myofilin and may correspond to the second region of relative sequence conservation between residues 75-106 in *Bombus*.

Video S4. Flightin and myofilin in context. Both the *Bombus* flightin (red) and myofilin (yellow) have multiple close contacts with myosin tails and curved layers as they pass over and through them. The movie depicts five curved layers colored starting from left to right: light gray, dark gray, white, light gray, dark gray. The light gray curved layers can be identified by the stub of density pointing downward that represents the end of the visible part of the proximal S2. The dark gray curved layers contain the C-terminus of the myosin coiled coil, which is pointing upward near the top of the frame. Flightin densities from *Bombus, Drosophila* and *Lethocerus* pass between a pair of myosin tails in the first dark gray curved layer before exiting the filament backbone. The *Lethocerus* flightin (red mesh) deviates from the *Bombus* structure immediately after exiting the backbone. The folded WYR domain contacts a myosin tail of the dark gray curved layer near its Skip 3 accommodation region (superimposed mesh) and its neighboring myosin tail within or near its assembly competence domain. Flightin makes an extensive contact with the innermost myosin tail of the first light gray curved layer. Flightin then passes over the white curved layer making a contact with myofilin and one myosin tail. It then passes between two myosin tails in the second light gray curved layer before terminating at the Skip 3 region (superimposed mesh) of the second dark gray curved layer. The C-terminus of flightin superimposes well with the so-called “blue density” from both *Lethocerus*, here depicted in blue mesh, and *Drosophila* (not shown). The N-terminal LKG domain of *Lethocerus* myofilin (yellow mesh) and *Bombus* myofilin (yellow solid) superimpose and are wedged between the first dark gray and the white curved layers. The second myofilin density of *Bombus* overlaps that of *Lethocerus* and appears wedged between the white and second light gray curved layers. The myofilin density from *Lethocerus* (yellow mesh) continues across the face of the white curved layer before terminating at the first dark gray curved. Note that the region between these two contacts with the first dark gray curved layer of *Lethocerus* myofilin, sandwich the Skip 2 region [21].

Video S5. Relationship between three non-myosin densities. Three non-myosin proteins in *Bombus* have intimate connections within the core of the thick filament. Note that the purple densities which represent the paramyosin core are unlikely to be an accurate representation of the paramyosin coiled coils because the paramyosin core does not conform to the helical arrangement of myosin tails. However, their radial positions are likely to be representative of the radial positions of paramyosin. The LKG domain at the N-terminus of myofilin (yellow) does not itself contact the paramyosin core, but a loop coming off the LKG domain extends towards and penetrates the paramyosin core. The loop in the WYR motif of flightin extends inward sufficiently to contact the paramyosin core, but none of the other flightin domains has that property though they could conceivably contact the non-helical part of paramyosin.

Video S6. Atomic modeling of flightin. Interpretation of the myofilin (yellow) and flightin (red) densities is ambiguous because the densities intersect within the long tail region. The predicted atomic model by AlphaFold2 using the *Bombus vosnesenskii* flightin sequence fit well within the “WYR” region and for the α-helix on its C-terminal side where the flightin and myofilin densities

intersect supporting the interpretation that the density extension after the WYR motif is part of flightin. The AlphaFold2 model is colored in such a way that the regions with higher confidence are colored with “cool” colors (purples, blues and greens) and the regions with low confidence are colored with “hot” colors (reds, oranges and yellows). The AlphaFold2 model on the N-terminal side of the WYR motif has low confidence and little secondary structure. This segment was therefore built *de novo*. On the C-terminal side, the AlphaFold2 model was also modified and rebuilt manually as polyalanine, which fit the density up to the C-terminus. The polyalanine model was then mutated to the flightin sequence. Interestingly, this elongation at the C-terminus overlaps the *Lethocerus* and *Drosophila* “blue” proteins indicating that the “blue” protein in those reconstructions is part of flightin.

Video S7. Myofilin atomic model. The atomic model of *Bombus* myofilin generated by Alphafold2 using the sequence of *Bombus terrestris* myofilin only fits the globular LKG domain at the N-terminus, the rest of the model is hard to untangle and does not overlap the density. AlphaFold2 predicts that the N-terminal LKG domain consists of a helix-loop-helix motif.
